# Simvastatin mediates inhibition of exosome synthesis, localization and secretion via multicomponent interventions

**DOI:** 10.1038/s41598-019-52765-7

**Published:** 2019-11-08

**Authors:** Ankur Kulshreshtha, Swati Singh, Mohd Ahmad, Kritika Khanna, Tanveer Ahmad, Anurag Agrawal, Balaram Ghosh

**Affiliations:** 1 Molecular Immunogenetics Laboratory and Centre of Excellence for Translational Research in Asthma & Lung disease, CSIR-Institute of Genomics and Integrative Biology, Mall Road, Delhi, 110007 India; 2 Molecular medicine group, International Center for Genetic Engineering and Biotechnology Aruna Asaf Ali Marg, Delhi, 110 067 India; 3 Multidisciplinary Center for Advanced Research and Studies, Jamia Millia Islamia, New Delhi, 110025 India

**Keywords:** Extracellular signalling molecules, Exocytosis

## Abstract

Discovery of exosomes as modulator of cellular communication has added a new dimension to our understanding of biological processes. Exosomes influence the biological systems by mediating trans-communication across tissues and cells, which has important implication for health and disease. In absence of well-characterized modulators of exosome biogenesis, an alternative option is to target pathways generating important exosomal components. Cholesterol represents one such essential component required for exosomal biogenesis. We initiated this study to test the hypothesis that owing to its cholesterol lowering effect, simvastatin, a HMG CoA inhibitor, might be able to alter exosome formation and secretion. Simvastatin was tested for its effect on exosome secretion under various *in-vitro* and *in-vivo* settings and was found to reduce the secretion of exosome from various cell-types. It was also found to alter the levels of various proteins important for exosome production. Murine model of Acute Airway Inflammation was used for further validation of our findings. We believe that the knowledge acquired in this study holds potential for extension to other exosome dominated pathologies and model systems.

## Introduction

Exosomes are cell secreted membrane bound nano-vesicular structures that have been shown to modulate the function and phenotype of recipient cells^1^, via transfer of associated lipids, proteins, RNA, and DNA species^2,3^. Such contents vary with cell lineage and state, accounting for a wide range of reported effects^4^. For example, stem cell exosomes render protective effects, while^5^ cancer cell derived exosomes promote metastasis^6,7^. Pro-inflammatory role for exosomes has also been demonstrated in other pathological conditions^8–10^. some studies have even proposed that strategies to reduce exosome secretion might have protective effects during inflammatory conditions^11,12^.

Formation and secretion of exosomes is a complex biological process, detailed knowledge of which remains incomplete. Though recent studies have started identifying key proteins involved in this process, such as PI3K, Akt, eNOS, Alix, syndecan, syntenin, Rab-27a and Rab-27b^12–14^, their precise role in this complex process is still under investigation. Other than proteins, ceramide and calcium have also been reported to regulate exosome biogenesis and secretion respectively^15,16^. Despite rapidly emerging evidence for associative role of exosomal communication in inflammatory diseases, there has been little progress towards identification of drug-candidates that can inhibit exosome secretion. While experimental studies have used siRNAs against important proteins^12^ and pharmacological inhibitors such as GW4869^16^, they still await approval for human use. Towards filling this lacuna, we reasoned if inhibition of cholesterol synthesis by statins could be a viable strategy for inhibiting exosome secretion, as cholesterol is most abundant component of exosomal membrane and statins represent safe and approved class of drugs for limiting cholesterol availability. This approach seemed plausible for three reasons: (a) cholesterol is a necessary lipid precursor for formation of exosomal membranes, and could offer a better target than proteins such as various Rab family proteins, whose exosome-independent functionality has not been explored yet, (b) although statins are mainly employed for cholesterol biosynthesis inhibition, they also have a number of poorly understood additional anti-inflammatory effects^17^ and, (c) repurposing of an existing drug for exosome reduction would be far more fast and efficient toward clinical application than discovering novel drug candidates.

Here, we investigated if simvastatin could reduce formation and/ or secretion of exosomes, and whether this could offer protection against exosome mediated pro-inflammatory response in experimental models of asthma and *in-vitro* model of atherosclerosis. Our current data supports a novel mode of action for simvastatin in inhibiting both exosome formation and secretion that explains some poorly understood aspects of anti-inflammatory effects of statins and can be further utilized in several exosome-mediated inflammatory conditions.

## Results

### Simvastatin reduces exosome secretion *in vitro*

**Figure 1:**
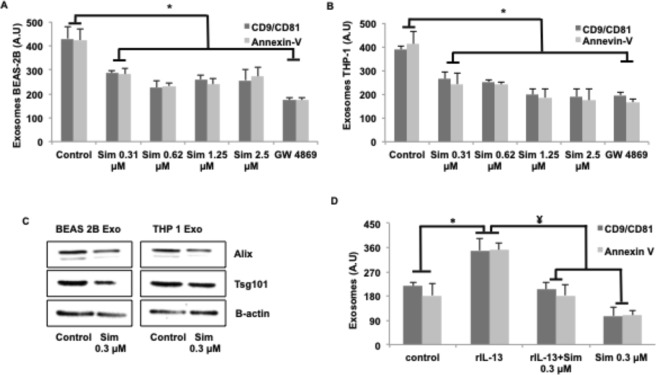
Simvastatin reduces exosomes secretion. **(A,B)**, Cells at a concentration of 2 × 10^6^/well of a 6-well plate were treated with indicated concentrations of simvastatin in 2 ml of media for a period of 24 hours, after which the culture supernatant was harvested and 1 ml from it was used for measuring exosomes. Secreted exosome levels in culture supernatant from simvastatin treated epithelial cells **(A)** and THP-1 monocytes **(B)**, measured as in Fig. S4 in the online supplement. (**C)**, Levels of exosome associated Alix, Tsg-101 and β-actin in pelleted exosome fraction from supernatant of 10^7^ simvastatin treated cells. **(D)** Effect of simvastatin treatment on exosome associated CD9/CD81 and Annexin V in cell culture supernatant from IL13 (25 ng/ml) and simvastatin treated epithelial cells. Data in (**A,B**,**D**) represent the mean ± SE from three independent experiments. Data in **C** is representative image from one of the two independent experiments. (_*_p < 0.05 vs Control and ^¥^p < 0.05 vs rIL13). Sim: Simvastatin.

Important role for cholesterol in formation of exosomes has been previously reported^16^, so we reasoned if limiting cholesterol availability in target cells could hinder exosome production. Literary evidences wherein cholesterol reduction has been shown to impair exocytosis of synaptic vesicles support this hypothesis^18^. To further test this hypothesis, we treated exosome-secreting cells with simvastatin, and measured effect on exosome secretion using a semi-quantitative fluorescent bead-based assay^10^ and for exosome associated proteins using western-blotting. We also validated the reduction in cholesterol levels upon simvastatin treatment (Fig. S1 in the online supplement). In an earlier report from our group, we have characterized these particles as being exosomes using EM and DLS for their size, which was found to be in the range of 40–200 nm and western blotting for having exosome specific proteins as CD63, Tsg101, HSC70 and Alix but we still validated the size and morphology for THP-1 and Beas-2B exosomes using TEM and DLS (Fig. S2 in the online supplement)^10^. Epithelial cells and monocytes treated with increasing concentration of simvastatin for a period of 24 hours exhibited a significant reduction in the level of secreted exosomes, as measured by the bead-based assay (Fig. 1A,B). A significant reduction of about 40% was noted at the 0.3 µM dose of simvastatin, which corresponds to non-toxic maximal plasma concentration associated with simvastatin therapy in humans^19^, confirming the plausibility of this effect at usual clinical dosing. The lack of toxicity was also confirmed by MTT staining (Fig. S3A in the online supplement) and visible inspection (Fig. S3B in the online supplement). The efficacy of bead-based assay was validated by confirming linearly increased detection of exosome-associated proteins, such as CD9/CD81 and Annexin-V, in cell-culture supernatants from increasing number of cells (Fig. S4 in the online supplement). These effects were confirmed further by measuring exosome-associated proteins, Alix, Tsg-101 and β-actin in pelleted exosome fraction of culture supernatant from lowest effective dose (0.3 µM) of simvastatin (Fig. 1C).

In an earlier study, we had demonstrated that IL-13 treatment led to increased production of pro-inflammatory exosomes from cultured Beas-2B cells^10^. We tested if simvastatin treatment could reverse this process as well, and observed that simvastatin treatment significantly reduced the levels of secreted exosomes from IL-13 treated Beas-2B epithelial cells (Fig. 1D), as measured by bead-based assay mentioned above. This corroborates well with previous observations wherein simvastatin has been shown to play a beneficial role in asthma^20,21^ and we propose that inhibition of proinflammatory exosomes could be one of the mechanisms behind simvastatin’s protective effects.

### Simvastatin reduces the intracellular levels of exosome-associated proteins

**Figure 2:**
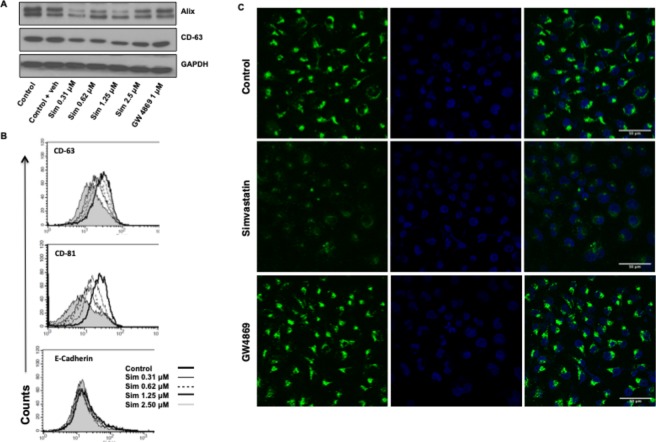
Simvastatin directly alters the level of various exosome associated proteins. (**A**) Western blots for Alix and CD63 levels in total cell protein treated with different doses of simvastatin. **(B)** Cell surface levels of CD63 and CD81 were measured by flow cytometry after treatment with various doses of simvastatin, E-cadherin was used as control surface marker. **(C)** Immunocytochemistry for CD63 on cells treated with 0.31 um of simvastatin for 24 hours. Sim: Simvastatin.

Our initial data suggested that simvastatin treatment led to reduction in exosome secretion, possibly by conventional action of cholesterol reduction by statin (Fig. S1 in the online supplement), but the detailed mechanism behind it remain unclear. Few reports have identified eNOS and Alix axis in exosome biogenesis wherein Alix has been shown to positively regulate the exosome secretory process^12,13^ and a negative regulatory role for eNOS^12^. We had previously demonstrated that simvastatin increases eNOS levels, both in cultured airway epithelial cells, as well as *in vivo*^22^, we further sought to determine if it was also affecting ALIX levels. We observed that simvastatin, but not another exosome inhibitor (GW4869), reduce the levels of Alix and CD-63 (Fig. 2A). Surface levels of important exosome associated proteins CD63 and CD81 but not E-cadherin, were also dose-dependently reduced by simvastatin (Fig. 2B), confirming the specificity and general reduction in these proteins by simvastatin treatment. The reduction in exosome-associated proteins like CD63 was additionally confirmed by immunofluorescence microscopy (Fig. 2C). Thus, reduction in levels of exosome synthesizing proteins may partly contribute to the simvastatin-mediated reduction in exosome secretion.

### The effect of simvastatin on exosome production and inflammation is only partially related to cholesterol reduction

**Figure 3:**
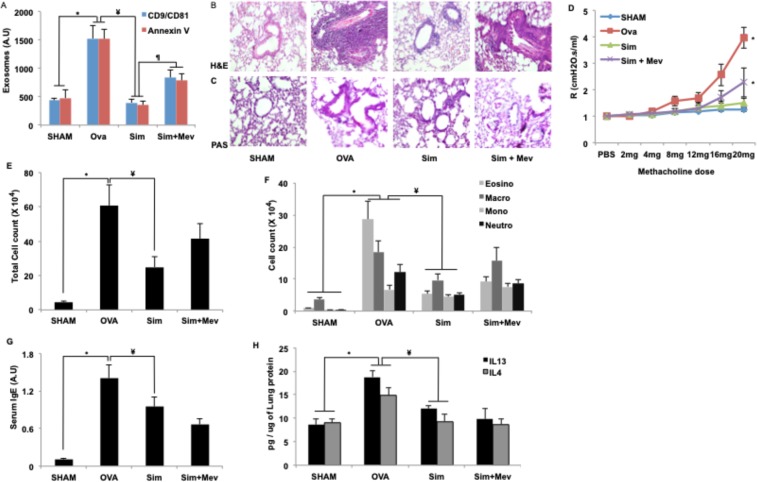
Effect of simvastatin and mevalonate cotreatment on inflammatory parameters. (**A**) Secreted exosome levels in BAL supernatant of mice from indicated groups. **(B,C)** Lung sections stained with hematoxylin and eosin (H&E, **B)** showing leukocyte infiltration, periodic acid–Schiff (PAS, **C)** for collagen deposition. **(D)** Airway resistance with increasing concentrations of methacholine 12 h after the last challenge. **(E,F)** Effect of indicated treatments on total leukocyte count **(E)** and differential leukocyte count enumerated by morphological criteria **(F)**. **(G)** Ova specific serum IgE levels measured by ELISA. **(H)** Cytokines IL-13 and IL-4 measured in pulmonary homogenate. Sections in **(B,C)** shown at 20X magnification. **Br**, Bronchus. Results **(A,D,E,F,G,H)** are the mean ± SE for each group from two experiments with 4–6 mice in each group, (*p < 0.05 vs SHAM; ^¥^p < 0.05 vs OVA; ^¶^p < 0.05 vs Sim), Sim: Simvastatin (40 µmg/kg/dose), Mev: Mevalonate (20 mg/kg/dose).

We (and others) have previously reported elevated levels of pro-inflammatory exosomes in asthmatic lung as well as in Bronchoalveolar Lavage Fluid (BALF) from mice of experimental model of allergic airway inflammation (AAI). Pharmacological inhibition of these exosomes was found to provide protective effect in experimental asthma^10^. To determine whether simvastatin treatment would inhibit exosome secretion and in turn attenuate AAI, we used a well-established mouse model of AAI (Fig. S5 in the online supplement). Further, to determine whether the effect of simvastatin was due to inhibition of the mevalonate formation step of cholesterol biosynthesis, or an independent pleiotropic effect, we additionally administered excess mevalonic acid^23^ to a group of simvastatin-treated mice with AAI. Increased exosome content in BAL fluid in AAI was fully reversed by simvastatin treatment (Fig. 3A). However, a large residual effect of simvastatin was seen even after mevalonate supplementation. This reduction in exosome content was found to be associated with protective effect on other asthmatic features as well, such as inflammation, mucin granule production, AHR, cell-count and serum IgE (Fig. 3B–G). Also, in ova-challenged mice, simvastatin significantly decreased the levels of IL-4, IL-13 (Fig. 3H), IL-5 and IL-10 (Fig. S6A,B in the online supplement). Mevalonate co-treatment, however, did not reverse the inhibitory effect of simvastatin on these cytokines. Levels of IFN-γ, an important Th1 cytokine, was unperturbed by any of the treatments (Fig. S6C in the online supplement). Thus the effect of simvastatin on exosomes and AAI in mouse model could only be partly explained by reduced cholesterol synthesis and may relate to other pleiotropic effects as suggested previously. As animal models are complex systems, often involving interaction of several components leading to less clean readouts, we further validated the exosome inhibitory potential of simvastatin in a simpler *in-vitro* interaction system mimicking pro-atherogenic exosomal interaction between monocytes and endothelial cells.

### Simvastatin mediated reduction in monocytic exosomes renders a protective effect in an *in vitro* model of atherosclerosis

Atherosclerotic plaque formation is a process whereby deposition of excess lipid and cholesterol in coronary artery leads to narrowing of blood vessels, thereby causing a reduction in blood flow to heart, resulting in heart failure. Atherosclerotic lesions are usually characterized by increased endothelial migration. In a study exploring this phenomenon^24^, authors implicated the role of exosomes (referred to as microvesicles in this paper) secreted by plaque-associated monocytes in endothelial migration. Microvesicle (MV) associated mir-150 was identified as the key driver of this process^24^. Since simvastatin has long been prescribed to patients of cardiovascular disorders, we wondered if one of the mechanisms by which it renders its protective effects could be by inhibiting microvesicle secretion from accumulated monocytes at plaque surface. For testing this hypothesis, we adopted the model previously described^24^, wherein monocytic microvesicles were shown to promote endothelial migration, and in turn atherosclerosis. These microvesicles contained several micro-RNA species including mir-150, mir-16 and mir-181a, however the pro-atherogenic nature of these vesicles was attributed majorly to mir-150, which caused reduction of c-myb in nearby endothelial cells, hence promoting their migration from the site of plaque formation.

**Figure 4:**
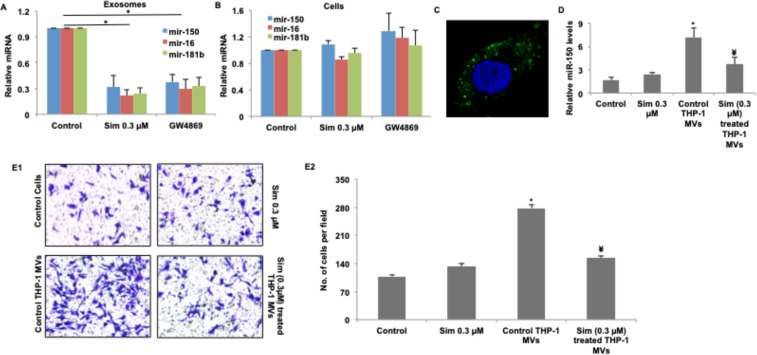
Simvastatin reduces exosome production from monocytes and attenuates exosome-enclosed mir-150 mediated endothelial cell migration. 1 × 10^6^ of THP-1 cells were seeded and treated with 0.3 µM of simvastatin and 1 µM GW4869 for a period of 24 hours. Cell pellet and supernatant was harvested and used separately for RNA isolation. Presence of indicated micro-RNAs was determined using qRT-PCR. Simvastatin mediated reduction of exosomes secretion from THP-1 monocytes results in lower levels of secretory miRNAs **(A)** but not intracellular miRNAs **(B**). (**C)** Uptake of DIO-labeled THP-1 derived exosomes (10 μg/mL) by HUVECs. **(D)** Relative mir-150 levels in HUVECs with indicated treatment (**E**), Simvastatin mediated reduction in exosome secretion by THP-1 monocytes results in lower mir-150 levels in HUVECs incubated with exosomes from simvastatin treated THP-1 in comparison to exosomes from same number of untreated THP-1 control cells, and consequent reduction in migration of endothelial cells. (*p < 0.05 vs Control in A, D and E2. ^¥^p < 0.05 vs Ctrl + MV in D and E2). Sim: Simvastatin, Control: Control HUVEC.

Simvastatin treatment of monocytic cell line, THP-1, led to reduction in exosome secretion (Fig. 1C), and a consequent reduction in the levels of secreted mir-150 (Fig. 4A). mir-16 and mir-181b were used as positive controls for exosomes-associated micro-RNA content. Simvastatin treatment however did not significantly alter the intracellular levels of any of these miRNAs (Fig. 4B). Incubation of THP-1 derived DIO labeled MVs with HUVECs led to rapid uptake of these vesicles by HUVECs (Fig. 4C), resulting in increased levels of mir-150 (Fig. 4D). Treatment of monocytes with simvastatin led to reduction in number of secreted microvesicles, and hence reduction in microvesicle-acquired mir-150 in HUVECs. mir-150 has been demonstrated to promote endothelial migration^24^ and we also observed similar phenomenon in HUVECs treated with THP-1 derived microvesicle, in presence or absence of serum as a chemoattractant (Figs 4E and S7 in the online supplement). Treatment of THP-1 with simvastatin before MV isolation significantly reduced migration of HUVECs, exhibiting an atheroprotective phenotype (Fig. 4E2). Our results thus suggest that inhibition of monocytic exosomes could be one of the alternate mechanism by which simvastatin renders a protective role in atherosclerosis.

### Simvastatin treatment alters MVB trafficking and results in their accumulation near the plasma membrane

**Figure 5:**
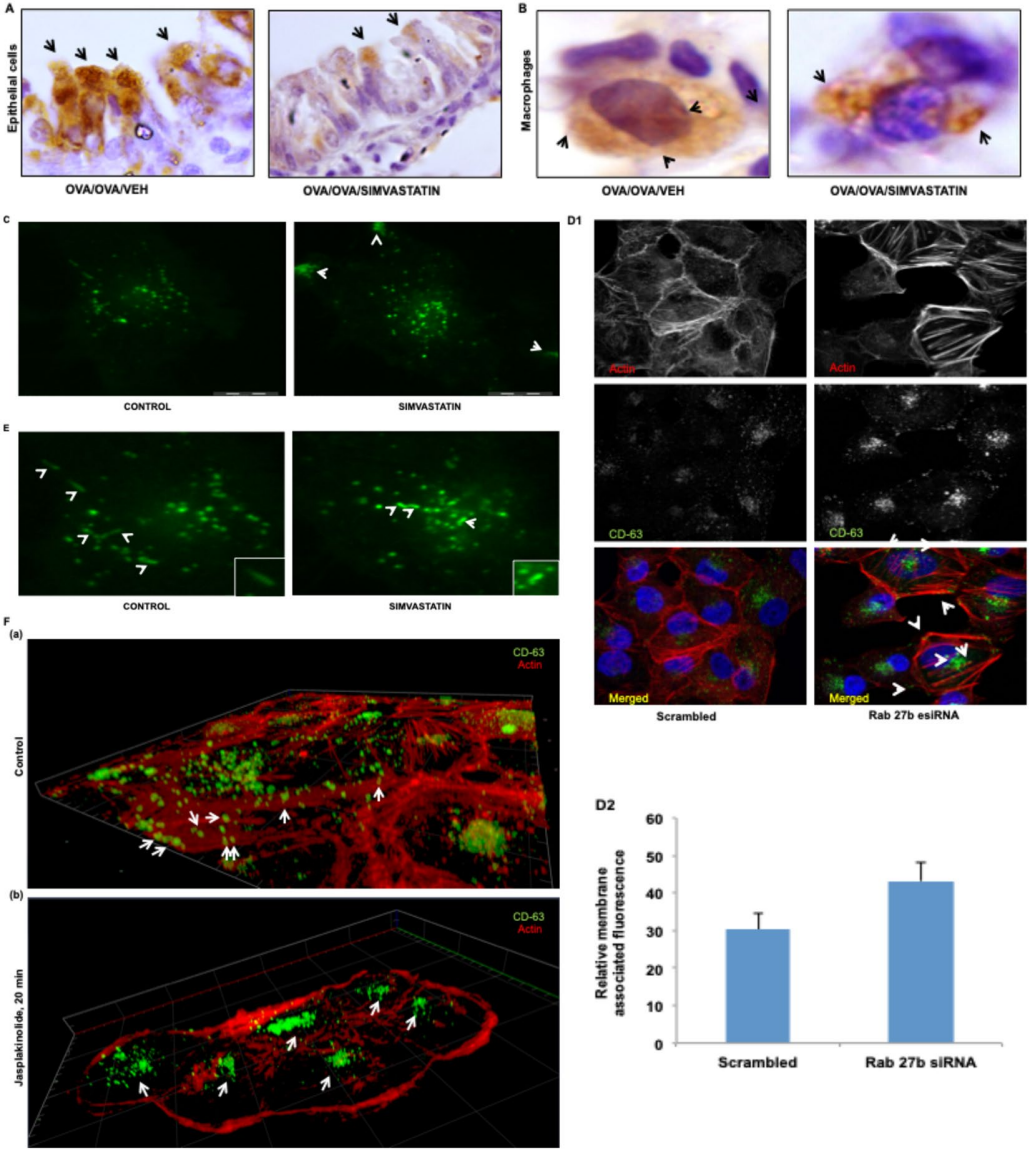
Simvastatin alters localization of CD63-positive compartments in cells. Representative Immunohistochemistry images for CD63 from lung tissue sections of OVA and OVA/Simvastatin treated mice in **(A)** epithelial cells and macrophages **(B)**. **(C)**, Representative TIRF images of CD-63 levels and localization post treatment with simvastatin in CD63-EGFP transfected cells. Arrows indicates CD63 localization pattern. **(D)** Distribution of CD63 in Rab-27b esiRNA treated cells **(D1)** and subplasmalemmal region associated CD-63 signals **(D2)**, as visualized by confocal microscopy. **(E)** Representative images showing CD63-EGFP distribution and its association with linear beeline like structures **(E1-E2, inset)** in control and simvastatin treated cells in subplasmalemmal region, detected by TIRF microscopy. (**F)** Localization of CD63 with actin in absence and presence of actin polymerization enhancer Jasplakinolide (100 nM). Images in **(E,F)** shown at 63X while **(A–D)** shown at 100 X magnification. Sim: Simvastatin.

Since we observed notable reduction in cellular CD-63 levels upon simvastatin treatment in *in vitro* systems (Fig. 2C), this led us to test if this observation extends to *in vivo* conditions as well. For this purpose, lung tissue sections from our mouse model of AAI were stained for CD-63. In AAI, lungs are known to have elevated exosome-associated proteins in epithelial cells and macrophages^10^. While inspecting lung-tissue sections of simvastatin treated mice, we observed an interesting phenomenon, wherein simvastatin treatment led to accumulation of CD-63 positive compartments near the plasma membrane in epithelial cells (Fig. 5A) as well as in macrophages (Fig. 5B).

Since we observed an accumulation of CD63 positive compartments near the plasma membrane, we sought to investigate the fate of these compartments by using TIRF microscopy of CD63-EGFP transfected cells. While we found uniform distribution of MVBs and normal movement pattern in control cells, simvastatin treated cells had accumulation of MVBs near plasma membrane and restricted movement of CD63 positive compartments, mostly towards the center of the cell (Fig. 5C, Movie 1 and Movie 2 in the online supplement).

Rab-27b is an important protein that has been shown to play important role in fusion of CD63+ve MVBs with plasma membrane with subsequent exosome release. When we knocked down Rab-27b using esiRNAs, we again observed similar accumulation of CD63+ve MVBs near plasma membrane that was higher in Rab-27b knocked-down cells in compare to scrambled control cells (Figs 5D1-D2 and S8B,C in the online supplement). We also found that simvastatin treatment led to reduction in the levels of Rab-27b (Fig. S8D in the online supplement).

Interestingly, while reviewing our TIRF data we noted several CD63 positive compartments aligning with each other in a beeline pattern during their movement, suggesting them to be associated with well-defined cytoskeletal structures (Fig. 5D inset). Actins tracks have recently been implicated in movement of Rab11 and CCL2 containing vesicles, which initiate actin nucleation and elongation for their movement in a directed fashion^25^. In light of this knowledge, we wanted to check if these CD-63 containing MVBs might also be utilizing the actin machinery for their movement. Closer examination of CD63 and actin inside cells revealed that the CD63 positive signals were indeed lying along the actin filaments and enhancing polymerization of actin structures by Jasplakinolide led to accumulation of CD-63 positive vesicles (Fig. 5F). Thus, we observe an association between the localization of CD-63 and actin, which may indicate that CD63 MVBs travel on actin tracks. Future studies in this direction could provide further insights into exosome biology.

## Discussion

Exosomes have recently come forth as important mediator of cellular communication governing progression of various inflammatory disorders, and their increasing relevance to human pathologies command better tools for understanding their biology as well as for therapeutic purposes. Here, we provide first evidence that cholesterol lowering drug simvastatin could inhibit exosome synthesis and trafficking. Our results also suggest that some well-known anti-inflammatory and atherosclerosis preventive effects of statins may be linked to inhibition of exosome secretion.

We had previously reported that exosomes actively play a proinflammatory role in asthma pathogenesis and speculated that molecules capable of reducing exosome secretion might play a protective role in asthma^10^. In this study, we report that simvastatin mediated exosome reduction indeed result in protective phenotype in murine model of asthmatic airway inflammation, which is also supported by recent reports of beneficial role of statins in human subjects^26,27^ and other experimental studies that focused on nitric oxide metabolism^22^. However, such *in vivo* models are complex and it is difficult to know whether the reduction in exosome secretion led to reduction in inflammation or *vice versa*. In support of an exosome-mediated effect, we found that the culture supernatant from simvastatin treated monocytes was diminished in exosomes and pro-inflammatory exosomal miRNA content, and also lacked the ability to induce endothelial migration, however we acknowledge the limitation that in most exosome related experiments it is usually difficult to get rid of lipoproteins using conventional centrifugation protocols, which may alter some of the results, and hence, future methods should focus on addressing this lacuna.

While we found a number of interesting leads, the mechanisms by which statins potentially inhibit exosome secretion is not completely clear. Our finding that simvastatin regulated multiple proteins of exosome production machinery suggests the existence of a dedicated inter-connected protein network for exosomal production, managed by few key master regulators. While we started the study in the belief that inhibiting cholesterol synthesis may attenuate exosomal membrane formation, this seems too simplistic. Mevalonate supplementation was unable to restore exosome secretion in mouse lungs (Fig. 4A). Clearly, these data do not exclude the possibility that simvastatin may exert other functional effects through alternative pathways as well. We also understand that full potential of such discoveries can be exploited only in conjunction with development of tools for their selective targeting as well.

Our observation that exosome-containing MVBs interacts closely with actin networks and simvastatin treatment significantly alters the association and length of these linear structures along with their membrane association offers exciting new directions and tool to look for novel proteins regulating exosomes via altering MVB movements.

In summary, this study identifies simvastatin as a potential tool to target pathway of exosome biogenesis, and significantly extends the role of simvastatin than just being a cholesterol lowering drug to a potential adjuvant for exosome dominated pathologies.

## Materials and Methods

### Cell lines

Bronchial Epithelial cell line BEAS-2B and human epithelial carcinoma cell-line NCI H-1299 was procured from ATCC (Middlesex, UK). BEAS-2B was cultured in BEGM media supplemented with bullet kit from Lonza, THP-1 and H-1299 cells were maintained in RPMI 1640 supplemented with 10% FCS that was depleted of FBS exosomes by spinning it at 100,000 g for 12 hrs. HUVECs were isolated from human umbilical cord and were cultured in M199 media supplemented with ECGF (Sigma, USA). Experiments with Human umbilical cords were performed as per guidelines and protocols approved by the CSIR-IGIB Human Ethics Committee. Prior informed consent from volunteers was obtained for the collection of material for this study.

### Animals

Male BALB/c mice (8–10 weeks old) were obtained from National Institute of Nutrition (Hyderabad, India) and acclimatized for a week prior to the experiments. All animals were maintained as per guidelines and protocols approved by the CSIR-IGIB Animal Ethics Committee.

### Antibodies

CD-63, Alix, Tsg-101, Hsp-70, β-actin and GAPDH were purchased from Santacruz Biotechnology (Santa Cruz, CA). Fluorescently labeled Annexin-V, CD-81 (FITC), CD-63 (FITC), CD-63 (PE) and CD-9 (FITC) were purchased from BD biosciences (San Jose, CA).

### Development of OVA-sensitized mouse model of asthma and treatment of mice with simvastatin

Mice were sensitized and challenged as described earlier^28^ (Fig. S5 in the online supplement). Mice were divided into four groups as indicated, each group (n = 6) was named according to sensitization/challenge/treatment: SHAM/PBS/VEH (normal controls, VEH-vehicle) called ‘SHAM’, OVA/OVA/VEH (allergic controls, OVA, chicken egg ovalbumin, Grade V, Sigma, USA) called ‘OVA’, OVA/OVA/Simvastatin (allergic mice treated with 40 mg/kg/dose Simvastatin, Sigma, USA) called ‘Statin’ and OVA/OVA/Simvastatin + Mevalonate (allergic mice treated with 40 mg/kg/dose Simvastatin and 20 mg/kg/dose mevalonate Sigma, USA) called ‘Mevalonate’ respectively.

### BAL isolation

24 h after last challenge, the mice were sacrificed using an overdose of sodium pentothal (100 mg/kg, i.p.). BAL was performed by tracheal cannulation with total 2 ml of PBS and around 1.5 ml of BAL fluid was recovered after 4 lavages.

### Exosome isolation

Exosomes were isolated using a series of centrifugation and ultracentrifugation techniques as described elsewhere^26^ with the modification wherein the collected media was spin at 2000 g for 15 minutes at 4 C, collected supernatant was subjected to 10,000 g at 4 C for 30 minutes, supernatant from 10,000 g fraction was filtered with a 0.2 μm membrane before subjecting it to ultracentrifugation at 100,000 g for 2 hours in a SW32-Ti swinging bucket rotor from Beckman coulter. The pellet was then washed with PBS by resuspending in 1 ml of PBS and adding 35 ml of PBS in the same tube to make-up the volume and re-pelleting the exosomes by spinning at 100,000 g for 1 hr. The pellet obtained was then suspended in 200 μl of PBS and layered on a continuous sucrose density gradient (0.32–2.5 M sucrose, 20 mM HEPES, pH 7.2) and centrifuged overnight at 100,000 g. Following day, fractions corresponding to density 1.08–1.21 were pooled and diluted with PBS to make up the volume to 35 mL and pelleted as pure exosomes, which was then used for transfer experiments. The exosome pellet was suspended in Laemmle buffer when used for western blotting.

### Semi-quantitative detection of exosomes by bead-based assay

For semi-quantitative detection of exosomes, antibody coated latex-aldehyde beads, 4um (Thermo Fisher Scientific) were used as described earlier^14^. Briefly, 20,000 anti-CD-63 antibody coated latex-aldehyde beads were washed in 2% BSA and then incubated overnight with 10,000 g supernatant of BALF or culture supernatant prepared as mentioned above. Briefly, BALF or culture supernatant was spin at 2000 g for 15 minutes at 4 C, collected supernatant was subjected to 10,000 g at 4 C for 30 minutes, supernatant from 10,000 g fraction was filtered with a 0.2 μm membrane before incubating with anti-CD-63 antibody coated latex-aldehyde beads. Next day, the bead bound exosomes were detected using surface proteins for exosomes or phosphatidylserine on their surface, using flow cytometer.

### Flow cytometry

For surface labeling, cells were incubated with the antibodies diluted 1:25 in staining buffer for 30 minutes on ice, followed by a PBS wash, after which the cells were fixed with 2% paraformaldehyde. Post fixation, cells were washed twice with staining buffer, resuspended in 200 ul of staining buffer and acquired on a BD FACSCalibur.

### Western blot

Protein extracts (30 μg) from control and simvastatin treated cells were analyzed by Western blot. Proteins were separated by 10% SDS-PAGE, and electroblotted onto nitrocellulose membranes (GE Healthcare). Nitrocellulose membranes were blocked with 5% Milk in PBS buffer with 0.1% v/v Tween-20 for 2 h at room temperature. Immunoblotting was performed with the goat polyclonal antibody, anti-CD-63, anti-Alix, anti-Tsg-101, anti-Hsp-70, anti-β-actin and anti-GAPDH were purchased from Santacruz Biotechnology (Santa Cruz, CA) and used at 1:1000 dilution in milk/PBS-T 1% w/v followed by incubation with secondary antibody at a dilution of 1:10000. Immunoblots were detected using HRP-conjugated secondary antibodies and enhanced chemiluminescence (Thermo Scientific Pierce ECL kit). The resulting Western blot images were scanned using Chemi Doc software (Biorad, Hercules, CA, USA).

### Measurement of Airway hyper-responsiveness (AHR)

AHR in the form of airway resistance was estimated in anesthetized mice using the FlexiVent system (Scireq, Canada) which uses a computer-controlled mouse ventilator and integrates with respiratory mechanics as described previously^25^. Final results were expressed as airway resistance with increasing concentrations of methacholine.

### Lung histology

Formalin-fixed, paraffin-embedded lung tissue sections were examined for airway inflammation, goblet cell metaplasia and sub-epithelial fibrosis with Hematoxylin & Eosin (H&E), Periodic acid-Schiff (PAS) and Masson-Trichrome (MT) staining respectively as described previously^28^. Briefly, grades of zero to four were given for no inflammation (zero), occasional cuffing with inflammatory cells (one), when most bronchi or vessels were surrounded by a thin layer (1–2 cells) (two), a moderate layer (3–5 cells) (three), and a thick layer (more than 5 cells deep) (four) of inflammatory cells and an increment of 0.5 was given if the inflammation fell between two grades and total inflammation score was calculated by addition of both peribronchial and perivascular inflammation scores.

### Measurements of cytokines in lung homogenate

Lung homogenates were used for ELISA of IL-4, IL-5, IFN-γ, IL-10 (BD Pharmingen, San Diego, CA) and IL-13 (R&D systems, Minneapolis, MN) as per the manufacturer’s protocol. Results were expressed in picograms and normalized by protein concentrations.

### Immunohistochemistry

Paraffin embedded tissue sections were used for preparation of 5μm tissue slides and immunohistochemistry was performed as described in^10^. CD-63 antibody was used at a dilution of 1:100.

### Immunofluorescence

Cells were seeded onto 0.17 mm coverslips and immunofluorescence was performed as described^28^, briefly, coverslips containing cells were washed twice with PBS, followed by incubation with 250 ul of BD Cytofix/Cytoperm solution for 20 minutes at 4 C. Anti-CD-63 antibody (FITC-conjugated) was diluted in BD Perm/Wash buffer at a concentration of 1:250 when used for staining CD63 alone, or with Alexa Fluor™ 594 Phalloidin (5 um final concentration), when used for simultaneous detection of CD-63 and F-actin and incubated on ice for 30 minutes. After which coverslips were washed twice with PBS, mounted with antifade reagent containing DAPI and observed under confocal microscope.

For exosome uptake assay by HUVECs, exosomes isolated from THP-1 cells were labeled with DiO-C16 for 1 hour and then unlabeled dye was removed by washing with PBS. Purified labeled exosomes were isolated by floating DIO labeled exosomes on sucrose density gradient. THP-1 exosomes thus isolated were resuspended in M-199 medium and incubated with cultured HUVEC cells. After incubation for 4 hours, HUVEC cells were washed, fixed, and observed under confocal microscopy.

### Quantitative polymerase chain reaction protocol

Real Time PCR for microRNAs were performed with sybr-green using custom primers. Equal concentration of starting RNA was used from each treatment for measuring micro-RNA in supernatant, while micro-RNA in cells were normalized to 18 s rRNA as internal control. Primer sequences used were mir-150 RT primer 5′-CTCAACTGGTGTCGTGGAGTCGGCAATTCAGTTGAGCACTGGTA-3′, mir-150 forward primer, 5′-ACACTCCAGCTGGGTCTCCCAACCCTTGTA-3′, mir-16 RT primer 5′-CTCAACTGGTGTCGTGGAGTCGGCAATTCAGTTGAGCGCCAATA-3′, mir-16 forward primer, 5′-ACACTCCAGCTGGGTAGCAGCACGTAAATA-3′, mir-181 RT primer, 5′-CTCAACTGGTGTCGTGGAGTCGGCAATTCAGTTGAGACCCACCG-3′, mir-181 forward primer 5′-ACACTCCAGCTGGGAACATTCATTGCTGTCG-3′, universal reverse primer 5′-GTGTCGTGGAGTCGGCAATTC-3′.

### HUVECs transmigration assay

The migration ability of HUVEC was tested in a Transwell Boyden Chamber (6.5 mm, Costar). The polycarbonate membranes (8 μm pore size) on the bottom of the upper compartment of the Transwells were coated with 1% gelatin matrix. Cells were suspended in serum-free M-199 culture medium at a concentration of 4 × 10^5^ cells/ml, treated with or without simvastatin treated THP-1 MVs for 2 hr and then added to the upper chamber (4 × 104 cells/well). Simultaneously, 0.5 ml of M-199 with 10% FBS was added to the lower compartment, and the Transwell-containing plates were incubated for 4 hr in a 5% CO_2_ atmosphere saturated with H_2_O. At the end of the incubation, cells that had entered the lower surface of the filter membrane were fixed with 90% ethanol for 15 min at room temperature, washed three times with distilled water, and stained with 0.1% crystal violet in 0.1 M borate and 2% ethanol for 15 min at room temperature. Cells remaining on the upper surface of the filter membrane (nonmigrant) were scraped off gently with a cotton swab. Images of migrant cells were captured by a photomicroscope. Cell migration was quantified by blind counting of the migrated cells on the lower surface of the membrane, with five fields per chamber.

### Statistical analysis

Data are expressed as mean ± standard error (SE). Significance of differences between groups was estimated using unpaired Student t-test for two groups or ANOVA with post hoc testing and Bonferroni correction for multiple group comparisons. Statistical significance was set at p ≤ 0.05.

## Supplementary information

Supplementary Information

Supplementary Movie 1

Supplementary Movie 2
