# Supplementary material for: Simvastatin mediates inhibition of exosome synthesis, localization and secretion via multicomponent interventions
Source: Sci Rep. 2019 Nov 8;9:16373. doi: 10.1038/s41598-019-52765-7 (PMC6841733; doi:10.1038/s41598-019-52765-7)
Supplement: Supplementary file 1 — Supplementary Information [file 41598_2019_52765_MOESM1_ESM.pdf]

# SUPPLEMENTARY INFORMATION

## Simvastatin mediates inhibition of exosome synthesis, localization and secretion via multicomponent interventions.

Ankur Kulshreshtha*[a,b], Swati Singh [a], Mohd Ahmad [a], Kritika Khanna [a], Tanveer Ahmad [c], Anurag Agrawal [a], and Balaram Ghosh*[a]

[a] Molecular Immunogenetics Laboratory and Centre of Excellence for Translational Research in Asthma & Lung disease, CSIR-Institute of Genomics and Integrative Biology, Mall Road, Delhi 110007, India.

[b] Molecular medicine group, International Center for Genetic Engineering and Biotechnology Aruna Asaf Ali Marg, Delhi 110 067, India

[c] Multidisciplinary Center for Advanced Research and Studies, Jamia Millia Islamia, New Delhi-110025, India

*To whom all correspondence should be addressed:

**Dr. Balaram Ghosh**

Molecular Immunogenetics Laboratory, CSIR-Institute of Genomics and Integrative Biology

Mall Road, Delhi 110007, India

Tel: 91-11-2766-2580

Fax: 91-11-2766-7471

E-mail: bghosh@igib.res.in

**Dr. Ankur Kulshreshtha**

Molecular medicine group, International Center for Genetic Engineering and Biotechnology

Aruna Asaf Ali Marg, Delhi 110 067, India

Tel: 91-11-2674-1317

Fax: 91-11- 2674-2316

E-mail: ankurk@icgeb.res.in

## SUPPLEMENTARY MATERIALS AND METHODS

### Cholesterol measurement

Cells were treated with indicated concentration of simvastatin for a period of 24 hours. Post treatment, media was removed and total lipids were extracted using a 3:2 mixture of hexane:2-propanol. The samples were dried under nitrogen, resuspended in 2-propanol and cholesterol estimation was performed using Cayman chemicals Cholesterol Fluorometric Assay Kit.

### Transmission Electron Microscopy

Purified exosomes resuspended in PBS were deposited on carbon-coated formvar grids. They were then negatively stained with uranyl acetate and washed with PBS to remove excess stain. The grids were dried and visualized on a FEI Tecnai Twin 20 electron microscope.

### Dynamic Light Scattering

Exosome samples resuspended in PBS were analyzed for their size on Malvern instrument.

**MTT Assay**

Beas-2B cells, 10,000 in nos. were seeded per well in triplicates/group in a 96-well plate. Following day, simvastatin treatment was given at indicated concentration. After 24 hours, media was removed and cells were incubated with MTT reagent (0.5mg/ml final concentration) in media for 3 hours. After that, the media was carefully removed and 100ul DMSO was added to solubilize the crystals and reading was taken at 570 nm.

**Quantification of subplasmalemmal region associated CD63 signals**

Cell boundaries were manually drawn around each cell as outer boundary and inner boundary (covering around 20% cytoplasmic area inside of the outer boundary). The signal corresponding to CD-63 in this region was calculated by subtracting signal of the inner area from total area. The values were later plotted to give the subplasmalemmal region associated CD63 signals for various treatment conditions.

## FIGURE LEGENDS

**Supplementary Figure S1. Effect of simvastatin on cellular cholesterol levels.** Cells at concentration of $2x10^6$/ well of a 6-well plate were treated with indicated concentrations of simvastatin in 2 ml of media for a period of 24 hours and cellular cholesterol was measured from different groups as mentioned in materials and methods. Figure plotted as mean ± SE from one of the two identical experiments performed in triplicate wells for each group.

**Supplementary Figure S2. Characterization of exosomes.** Exosomes from THP-1 **(A)** and Beas-2B **(B)** cells were characterized by TEM for their morphology and DLS for their size distribution.

**Supplementary Figure S3. Assessing effect of various doses of Simvastatin on health of Beas-2B cells.** Effect of indicated concentrations of simvastatin on cell viability was assessed by MTT assay **(A)** or visual inspection **(B)**. Figure in **(A)** plotted as mean ± SE from one of the two identical experiments performed in triplicate wells for each group.

**Supplementary Figure S4. Detection of bead-bound exosomes from different number of cells.** Beads incubated with same volume of culture supernatant from 0.25, 0.5 and 1.0 million cells, and then analyzed for exosome associated proteins by flow cytometer.

**Supplementary Figure S5. Development of murine model of asthma.** Protocol to induce allergic asthma in mice. Male BALB/c (8–10-week-old) mice were grouped, sensitized, and challenged. Simvastatin or Mevalonate was administered via intraperitoneal injections (i.p. inj.) from Days 21–28 (A), Measurements of lung function and sacrifice were performed on Day 28. AHR, airway hyperresponsiveness; OVA, chicken egg ovalbumin

**Supplementary Figure S6. Effect of simvastatin and mevalonate co-treatment on various cytokines. (A-C),** Levels of cytokines measured in total pulmonary homogenates from various groups, IL-5 **(A)** IL-10 **(B)** and IFN-γ **(C)**. Figures (A-C) are plotted as mean ± SE of two experiments with 4-6 mice in each group, * indicates $p<0.05$ vs OVA. Sim: Simvastatin (40 mg/kg/dose), Mev: Mevalonate (20 mg/kg/dose)

**Supplementary Figure S7. Simvastatin inhibits endothelial migration.** HUVECs treated with THP-1 MVs were seeded onto the top of 8μm pore size filter membrane with plain media in top chamber and plain media in bottom chamber.

**Supplementary Figure S8. Rab27b knockdown produces similar phenotype as simvastatin treatment. (A)** Knock down of rab27b using esi-RNA. **(B)** Schematic used for defining area to calculate subplasmalemmal region associated CD63 signals**. (C),** Knock down of rab27b results in accumulation of CD63 positive compartments as seen by simvastatin treatment.

**Movies.** Cells were transfected with CD-63 EGFP. 24 hours later, transfected cells were either left untreated **(Movie 1)** or treated with simvastatin for 12 hours **(Movie 2)**, and live-cell recordings were carried out using total-internal reflection fluorescence (TIRF) module equipped microscope.

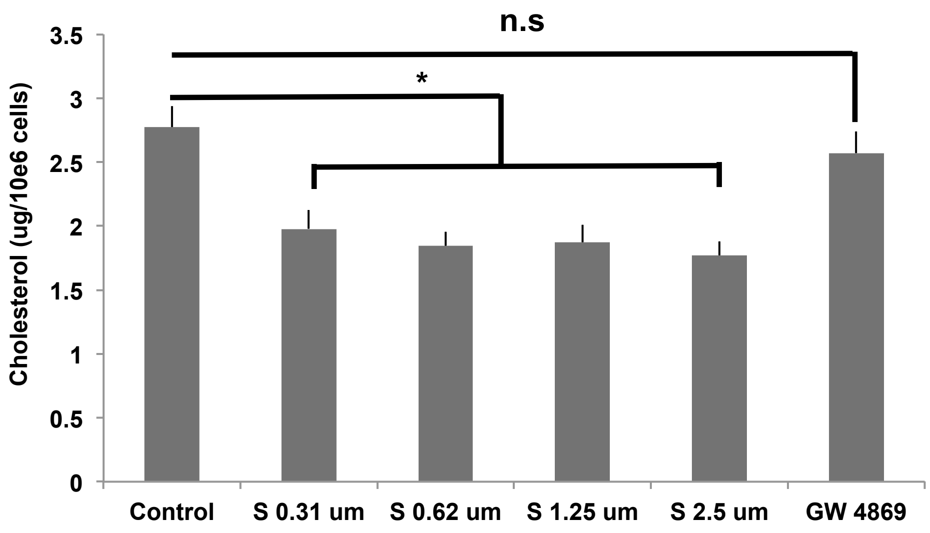


**Supplementary Figure S1.**

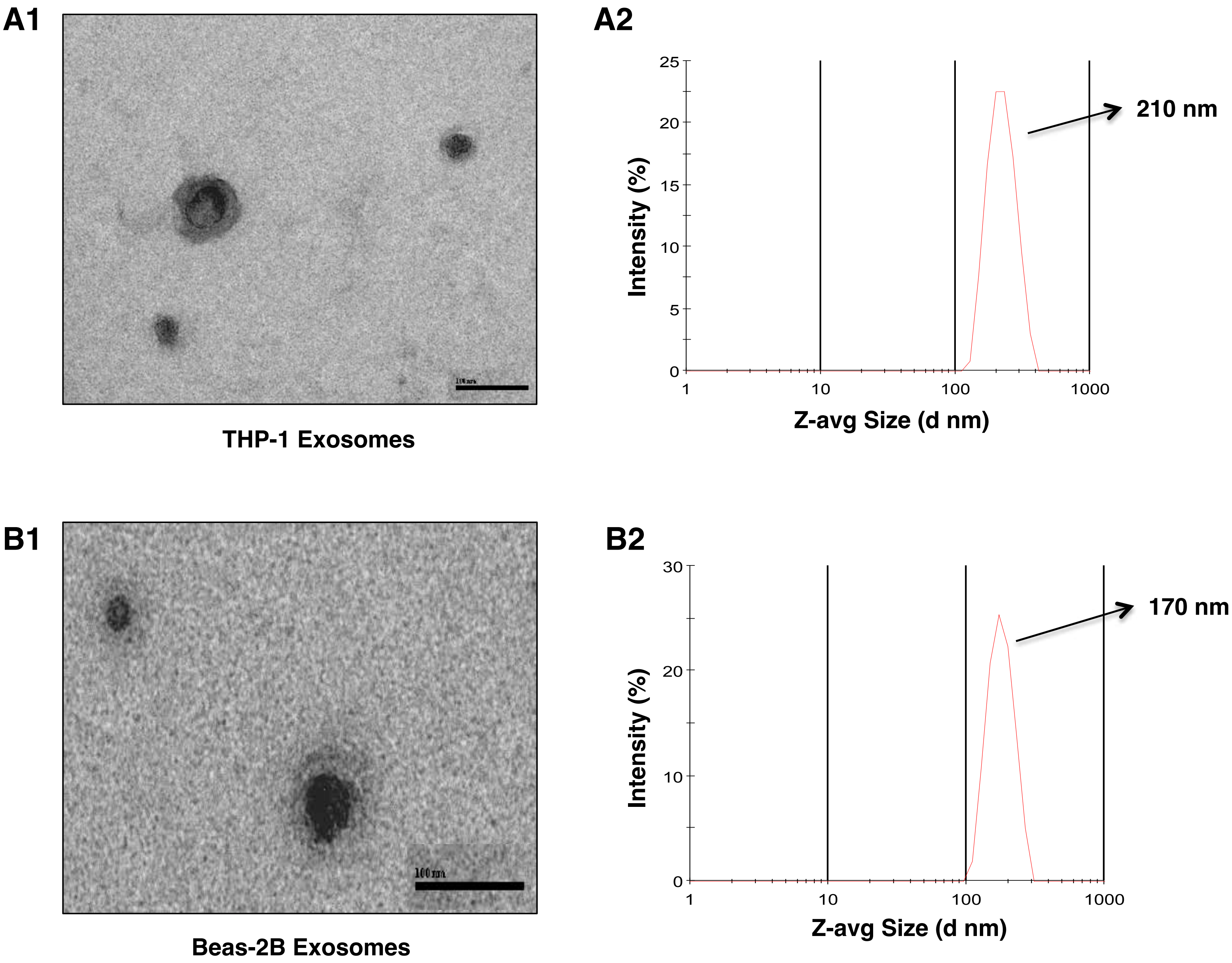


Supplementary Figure S2

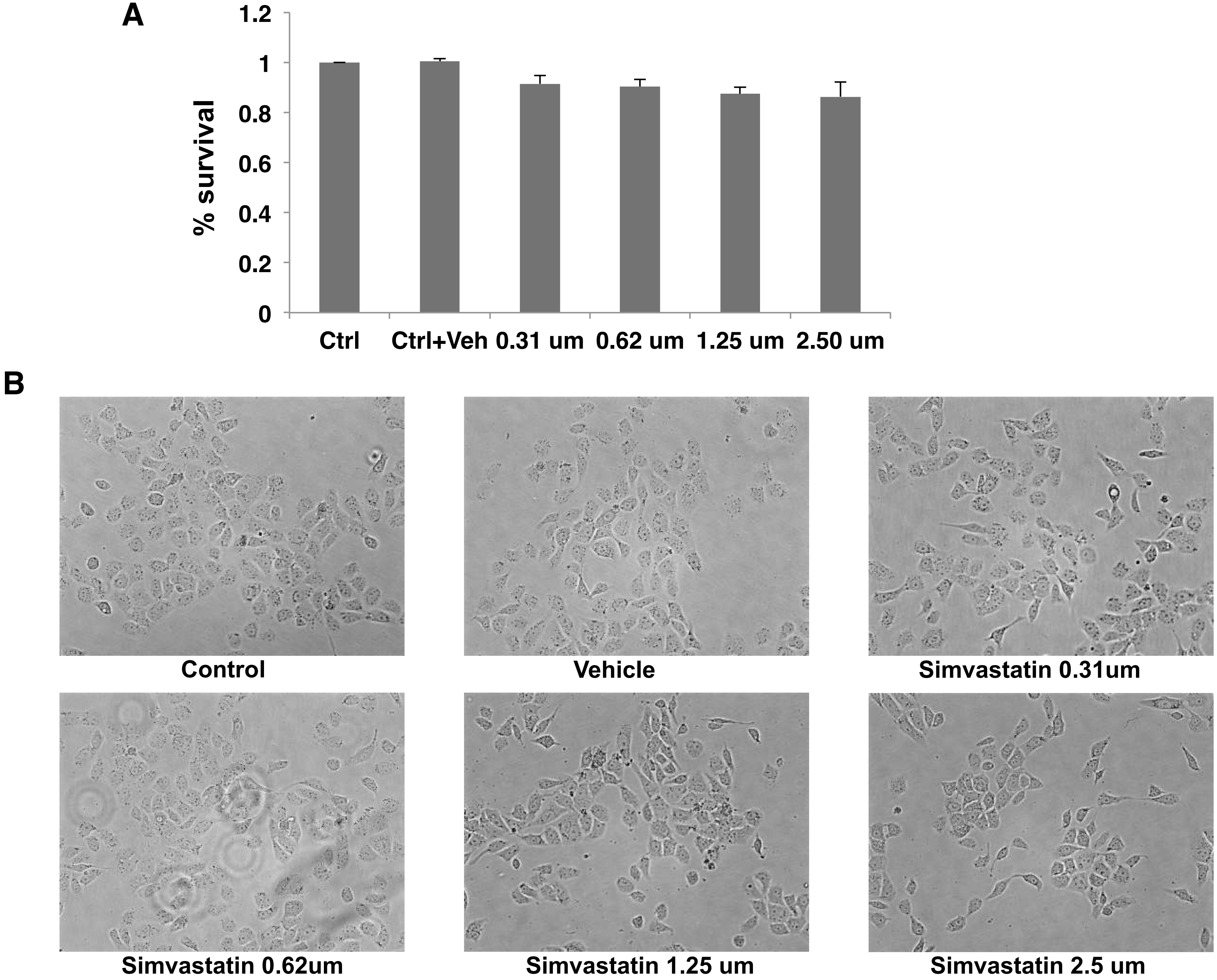


Supplementary Figure S3

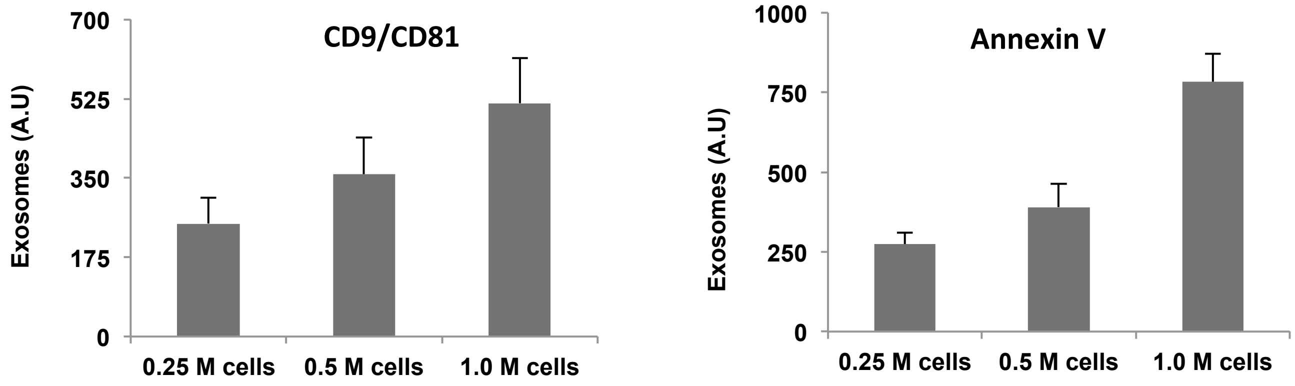


Supplementary Figure S4

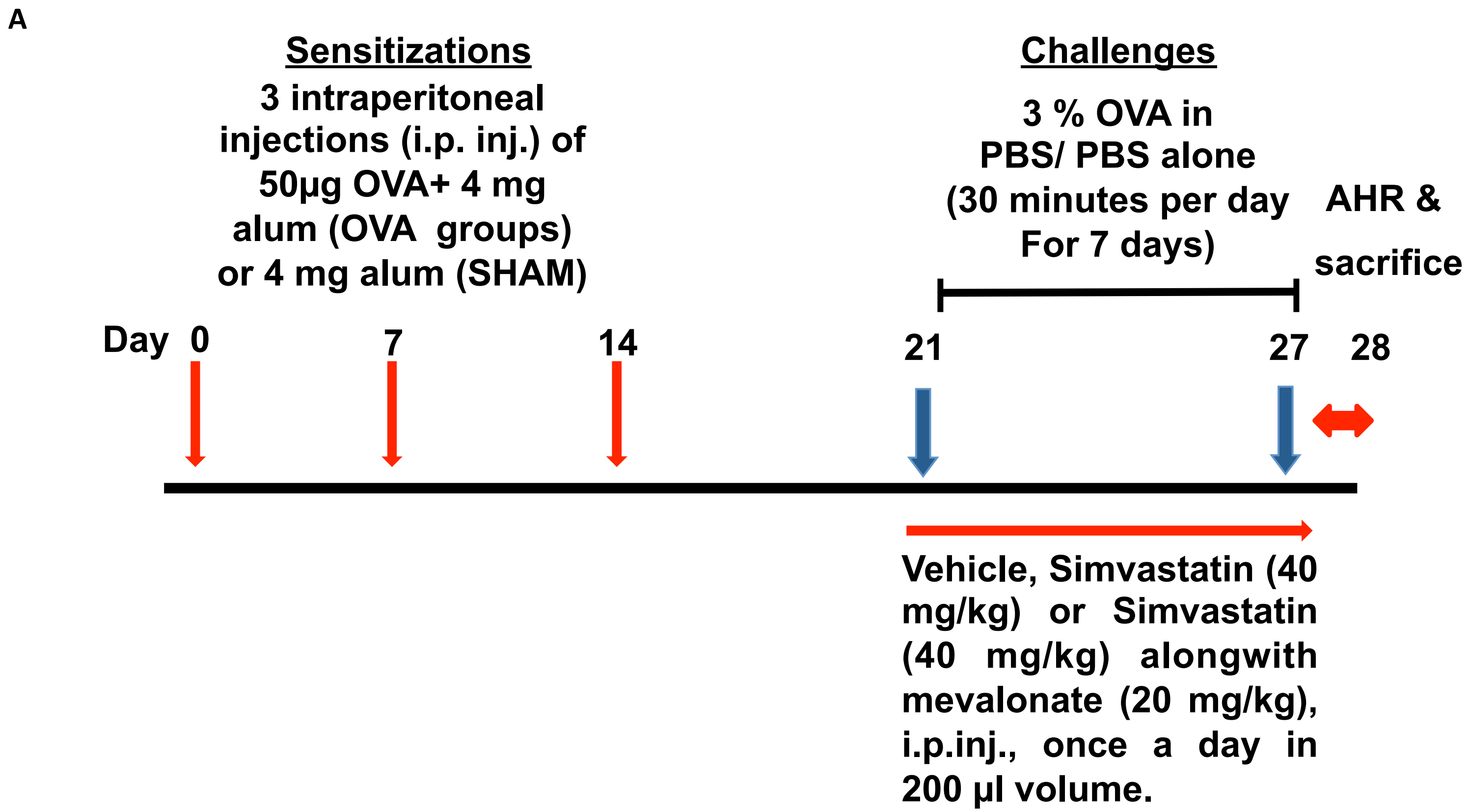


Supplementary Figure S5

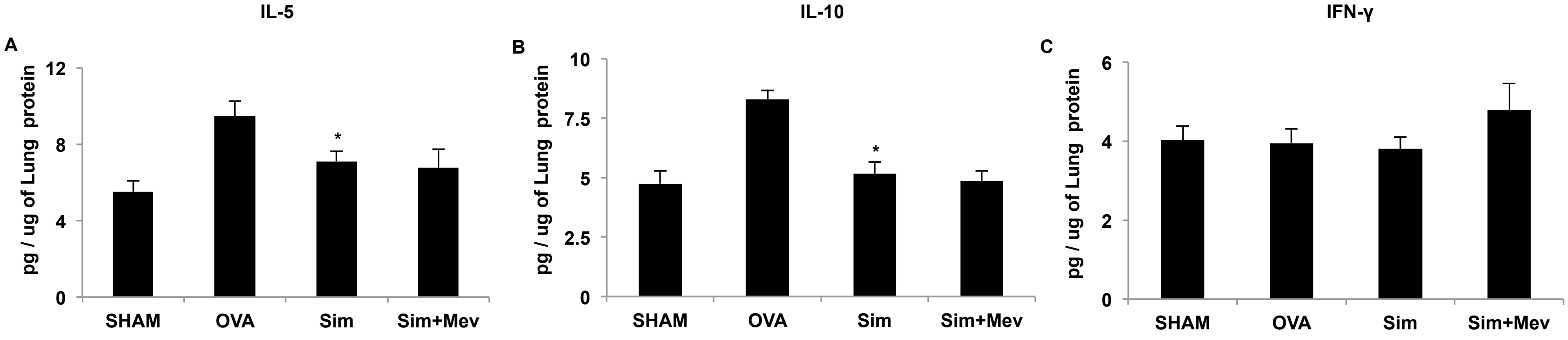


**Supplementary Figure S6**

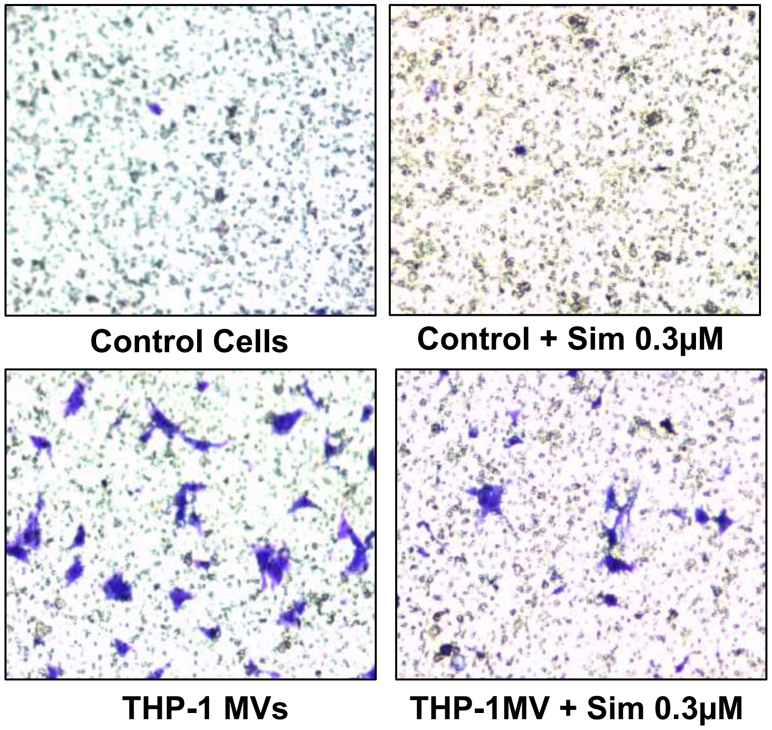


**Supplementary Figure S7**

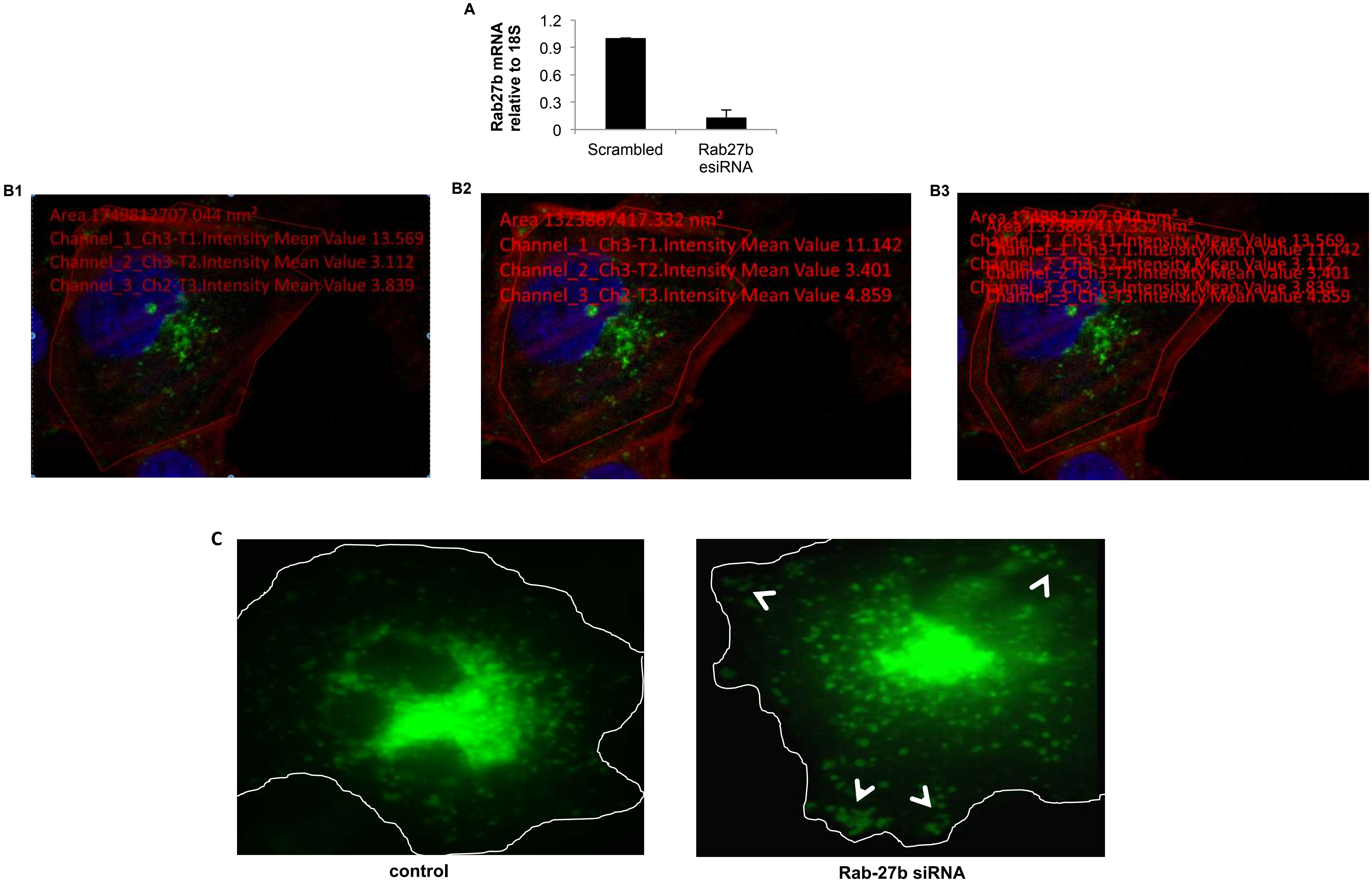


**Supplementary Figure S8**
